# Virus strain from a mild COVID-19 patient in Hangzhou represents a new trend in SARS-CoV-2 evolution potentially related to Furin cleavage site

**DOI:** 10.1080/22221751.2020.1781551

**Published:** 2020-07-03

**Authors:** Xi Jin, Kangli Xu, Penglei Jiang, Jiangshan Lian, Shaorui Hao, Hangping Yao, Hongyu Jia, Yimin Zhang, Lin Zheng, Nuoheng Zheng, Dong Chen, Jinmei Yao, Jianhua Hu, Jianguo Gao, Liang Wen, Jian Shen, Yue Ren, Guodong Yu, Xiaoyan Wang, Yingfeng Lu, Xiaopeng Yu, Liang Yu, Dairong Xiang, Nanping Wu, Xiangyun Lu, Linfang Cheng, Fumin Liu, Haibo Wu, Changzhong Jin, Xiaofeng Yang, Pengxu Qian, Yunqing Qiu, Jifang Sheng, Tingbo Liang, Lanjuan Li, Yida Yang

**Affiliations:** a Department of Gastroenterology, the First Affiliated Hospital, College of Medicine, Zhejiang University, Hangzhou, People’s Republic of China; b Emergency and Trauma Center, the First Affiliated Hospital, College of Medicine, Zhejiang University, Hangzhou, People’s Republic of China; c Center of Stem Cell and Regenerative Medicine, and Bone Marrow Transplantation Center of the First Afﬁliated Hospital, Zhejiang University School of Medicine, Zhejiang Engineering Laboratory for Stem Cell and Immunotherapy, Institute of Hematology, Dr. Li Dak Sum & Yip Yio Chin Center for Stem Cell and Regenerative Medicine, Hangzhou, China; d State Key Laboratory for Diagnosis and Treatment of Infectious Diseases, National Clinical Research Center for Infectious Diseases, Collaborative Innovation Center for Diagnosis and Treatment of Infectious Diseases, Department of Infectious Diseases, The First Affiliated Hospital, College of Medicine, Zhejiang University, Hangzhou, People’s Republic of China; e State Key Laboratory for Diagnosis and Treatment of Infectious Diseases, National Clinical Research Center for Infectious Diseases, Collaborative Innovation Center for Diagnosis and Treatment of Infectious Diseases, The First Affiliated Hospital, College of Medicine, Zhejiang University, Hangzhou, People’s Republic of China; f Department of Colorectal Surgery, the First Affiliated Hospital, College of Medicine, Zhejiang University, Hangzhou, People’s Republic of China; g Laboratory Department, the First Affiliated Hospital, College of Medicine, Zhejiang University, Hangzhou, People’s Republic of China; h Department of Neurosurgery, the First Affiliated Hospital, College of Medicine, Zhejiang University, Hangzhou, People’s Republic of China; i Department of Hepatobiliary and Pancreatic Surgery, the First Affiliated Hospital, College of Medicine, Zhejiang University, Hangzhou, People’s Republic of China

**Keywords:** SARS-CoV-2, COVID-19, Furin, ACE2

## Abstract

The mutations in the SARS-CoV-2 virus genome during COVID-19 dissemination are unclear. In 788 COVID-19 patients from Zhejiang province, we observed decreased rate of severe/critical cases compared with patients in Wuhan. For mechanisms exploration, we isolated one strain of SARS-CoV-2 (ZJ01) from a mild COVID-19 patient. Thirty-five specific gene mutations were identified. Phylogenetic and relative synonymous codon usage analysis suggested that ZJ01 may be a potential evolutionary branch of SARS-CoV-2. We classified 54 global virus strains based on the base (C or T) at positions 8824 and 28247 while ZJ01 has T at both sites. The prediction of the Furin cleavage site (FCS) and sequence alignment indicated that the FCS may be an important site of coronavirus evolution. ZJ01 mutations identified near the FCS (F1-2) caused changes in the structure and electrostatic distribution of the S surface protein, further affecting the binding capacity of Furin. Single-cell sequencing and ACE2-Furin co-expression results confirmed that the Furin expression was especially higher in glands, liver, kidneys, and colon. The evolutionary pattern of SARS-CoV-2 towards FCS formation may result in its clinical symptom becoming closer to HKU-1 and OC43 caused mild flu-like symptoms, further showing its potential in differentiating into mild COVID-19 subtypes.

## Introduction

The outbreak of a novel coronavirus (SARS-CoV-2) and the associated disease (COVID-19) began in Wuhan, China, near the end of 2019. The disease quickly affected the whole country. COVID-19 continues to pose a severe threat to public health and economic prosperity in China [1,2]. Through a quick response and drastic measures that included quarantining Wuhan City beginning on 23 January 2020, the spread of SARS-CoV-2 in China was effectively controlled. However, its ensuing sporadic global appearance [3,4] and rapid dissemination in Japan, South Korea, Iran, and Italy resulted in the pandemic spread of SARS-CoV-2 [5–7]. Therefore, it is important to determine the clinical and virologic characteristics of SARS-CoV-2 during its dissemination.

An important and common feature of viruses, including SARS-CoV-2, is that their increased transmissibility is usually accompanied by decreased virulence, which is reflected in the disease trajectory. COVID-19 was more severe in Wuhan soon after its appearance, with severe/critical and fatality rates of approximately 32% and 11%, respectively [8,9]. Subsequent data as the disease spread revealed a milder form of COVID-19 in Zhejiang province [10] and nationwide [11]. On the other hand, the transmissibility increased from a basic reproductive number (R0) of 2.2 [12] and 2.68 [6] in Wuhan to 3.77 [13] at the national level. Furthermore, the observation of a similar viral load in symptomatic and asymptomatic COVID-19 patients revealed the capacity of SARS-CoV-2 for occult transmission [14].

Changes in the epidemiological and clinical features of COVID-19 relate to the virologic changes of SARS-CoV-2, in which the spike (S) surface envelope protein plays an important role [15]. Generally, its surface unit (S1) is responsible for host entry by binding to the cell receptor, while its transmembrane unit (S2) drives the fusion of viral and cellular membranes [16]. Therefore, it is valuable to focus on the sequence mutation and conformation change in S protein for SARS-CoV-2 evolution in an established model with the aim of explaining the related changes in COVID-19.

In this study, we identified a severe/critical rate of 9.9% in 788 confirmed COVID-19 patients in Zhejiang province, and a median of 11 days of positive nuclear acid in 104 patients from our hospital, indicating the tendency of COVID-19 progression towards a milder but more infective disease. Based on these clinical findings, we performed in-depth bioinformatics analysis by comparing the virologic features of 52 previously reported strains of SARS-CoV-2, including Bat CoV, SARS-CoV and SARS-CoV-2 in Wuhan and ZJ01. The latter was an isolate we described from a patient with mild COVID-19 in Zhejiang province. The evidence of continuous evolution of potential Furin cleavage sites (FCSs) on the S protein of SARS-CoV-2 suggests that Furin may play an important role in viral evolution. The establishment of a novel SARS-CoV-2 categorization system may facilitate our understanding of virus evolution and its influence on the severity and progression of COVID-19.

## Materials and methods

### Data sources and ethics

This retrospective study investigating the epidemiological, clinical and virologic features of COVID-19 was performed at designated hospitals in Zhejiang province between 17 January and 7 February 2020. We subsequently calculated the time period of positive COVID-19 nucleic acid in our hospital. All patients were diagnosed with COVID-19 according to the World Health Organization interim guidance [17] and the preliminary data were promptly reported to the authority of Zhejiang province. The study was approved by the Clinical Research Ethics Committee of the First Affiliated Hospital, College of Medicine, Zhejiang University (Approval No. IIT20200005C). Written informed consent was waived by the ethics committee of the participating hospitals, as the study involving an emerging infectious disease and was part of a continuing nationally authorized public health outbreak investigation. The subtypes of COVID-19 were categorized as mild, severe and critical, as recently described [11]. The period of positive nucleic acid is defined as the date of confirmed nucleic acid positivity minus the date of confirmed nucleic acid negativity.

### SARS-CoV-2 collection and confirmation

SARS-CoV-2 was confirmed from samples of throat swabs and sputum in our hospital and the Center for Disease Control and Prevention (CDC) facility in Zhejiang province using real-time RT–PCR targeting typical nucleic acids using a previously acknowledged protocol (Bio-germ, Shanghai, China) [8]. All patients underwent chest radiography or computed tomography (CT) scan on admission. Other respiratory viruses including influenza A (H1N1, H3N2, and H7N9), influenza B, respiratory syncytial virus, SARS-CoV, and Middle East Respiratory Syndrome (MERS)-CoV were excluded. Epidemiological, anthropometric, clinical, and laboratory data were collected on admission, with specific attention paid to the period between symptom onset and outpatient visit/PCR confirmation/hospital admission. One strain of SARS-CoV-2 was successfully isolated from a single sputum sample of a patient with a mild COVID-19 case at the time of admission in our hospital. The sample was sent to The Beijing Genomics Institute (BGI) company for whole genome sequencing using a previously reported method [18]. Briefly, 200 μL of the virion suspension was frozen and thawed three times. A 140 μL aliquot of the final supernatant was used for RNA extraction using the QIAamp Viral RNA Mini Kit (52904; Qiagen, Hilden, Germany) according to the manufacturer’s recommendations. The qualified double-stranded DNA library was sequenced with PE150 using the Novaseq 6000 platform (Illumina, San Diego, CA, USA).

### SARS-CoV-2 sequence data collection and alignment

Currently, available coronavirus sequences (*n* = 85) were obtained from the NCBI viral genome database (https://www.ncbi.nlm.nih.gov/, *n* = 65), Genome Warehouse (https://bigd.big.ac.cn/gwh/, *n* = 12), CNGBdb (https://db.cngb.org/, *n* = 3), and NMDC (http://nmdc.cn/#/coronavirus, *n* = 6). The sequence of ZJ01 (BataCov/Zheji ang/ZJ01/2019) was previously reported by us. The 52 SARS-CoV-2 sequences were collected from China (*n* = 30), Japan (*n* = 5), Nepal (*n* = 1), South Korea (*n* = 1), Australia (*n* =1), Finland (*n* = 1), and the United States (*n* = 13) between 26 December 2019, and 5 February 2020. The Furin protein sequence was downloaded from the NCBI database. Multiple sequence alignment of all coronavirus genomes was performed using MEGA v7.0.26.

### Phylogenetic and relative synonymous codon usage analyses

Phylogenetic analysis was performed on a total of 80 coronavirus strains, covering six species (human, bat, mink, camel, rat, and pig). SARS-CoV-2 was acquired from 17 cities in seven countries from 23 December 2019 to 5 February 2020, which overlapped with the time period from virus outbreak to dissemination. The evolutionary history was constructed based on the coronavirus S protein by the neighbour-joining method. The bootstrap consensus tree inferred from 2000 replicates was used to represent the evolutionary history of the taxa analysed. Branches corresponding to partitions reproduced in <30% bootstrap replicates were collapsed. The evolutionary distances were computed using the Kimura 2-parameter method and expressed as the number of base substitutions per site. Evolutionary analyses were conducted in MEGA7 v7.0.26. Relative synonymous codon usage (RSCU) analysis was performed to compare the differences between 49 strains of SARS-CoV-2 and Homo. A heat map was drawn using MeV 4.9.0 software. All available coding sequences (minimum >28 kbp) were calculated with Codon W1.4.2.16, followed by inter-relationship calculation based on the Euclidean distance method.

### Mutation site analysis and prediction of Furin cleavage site (FCS)

SimPlot v.3.5.1.15 was used to analyse the potential genetic recombination. Visualization of the mutation site between RATG13 and ZJ01 was performed using Multalin software (http://multalin.toulouse.inra.fr/multalin/multalin.html). Multiple sequence alignment was applied using the Muscle (codons) function of MEAG v7.0.26. Genetic mutation sites were analysed using DNAMAN v9.0.1.116. The functional domain distribution of SARS-CoV-2 and S proteins was plotted using IBS v1.0.3. FCS prediction was carried out in ProP 1.0 Server (http://www.cbs.dtu.dk/services/ ProP/) [19] and is presented as Furin score (range 0–1). A score closer to 1 indicates a higher possibility of the existence of an FCS.

### Homology modelling and Adaptive Poisson-Boltzmann Solve (APBS) analysis

Target protein was downloaded from NCBI (https://www.ncbi.nlm.nih.gov/ protein/1791269092) and the corresponding homology models were predicted by SWWISS-MODEL (https://swwassmodel.expasy.org/). Protein sequence alignment and APBS analysis were performed using PyMOL v2.3.3 on an Intel i7 9700F processor. APBS was calculated and evaluated using PyMOL v2.3.3, as previously reported [20].

### Single-cell transcriptome data analysis

The raw counts or processed data were downloaded from the Tissue Stability Cell Atlas (https://www.tissuestabilitycellatlas.org/) and Gene Expression Omnibus (https://www.ncbi.nlm.nih.gov/). Lung, colon and liver data were obtained from the Tissue Stability Cell Atlas [21], GSE116222 [22], and HCA [23], including samples of lung (*n* = 5), colonic epithelium (*n* = 3) and hepatic tissues (*n* = 5) from healthy volunteers and organ donors. Lung and liver data were processed before downloading and were directly used for data analysis and visualization. For liver data, cells with <100 expressed genes and 1500 unique molecular identifier counts and >50% mitochondrial genome transcripts were removed. Genes expressed in fewer than three cells were also removed.

Normalization and principal component analysis (PCA) were performed using the R package Seurat [24], with different dataset-based data processing methods. For the liver, the first 40 principal components resulting in the PCA were used to perform cell clustering and nonlinear dimensionality reduction (uniform manifold approximation and projection, UMAP). For the colon, the R package Harmony [25] was used to remove batch effects with default settings. We used the first 40 components to perform cell clustering and nonlinear dimensionality reduction, similar to liver data. Depending on the expression level of cell markers provided in the original article corresponding to the single-cell RNA (scRNA)-seq datasets, we further estimated which cell types the cell clusters belonged to. Annotated clusters were then visualized using UMAP plots with the “DimPlot” function in Seurat. Normalized gene expression levels were presented in the UMAP and violin plots using the R package ggplot2 [26].

## Results

### Epidemiological and clinical characteristics of 788 enrolled COVID-19 and ZJ01 patients

As shown in Table 1, 51.65% of the 788 enrolled patients were males. The rate of smoking was low (6.85%). The three predominant co-existing conditions were hypertension (15.99%), diabetes (7.23%) and chronic liver disease (3.93%). The median period from illness onset to outpatient visit, PCR confirmation and hospital admission were 2, 4 and 3 days, respectively. The most common symptoms were fever (80.71%) and cough (64.21%). CT/X-ray evidence of disease was greatest for bilateral pneumonia (37.56%). The rates of mild, severe and critical types of COVID-19 were 90.1%, 7.74% and 2.16%, respectively. The ZJ01 patient was male and 30 years of age, with no histories of smoking or any co-existing condition. He visited outpatient clinics one day after symptom onset and was admitted to the hospital with COVID-19 on the same day following the PCR result. He had not been to Wuhan, and none of his family members have been virus positive at the time of writing. Consistent with other COVID-19 patients, his symptoms included fever, cough and sputum production, with bilateral pneumonia evident in the CT scan. The patient had mild type COVID-19, with normal results for routine blood parameters and inflammation markers (C-reactive protein and procalcitonin). The patients displayed elevated levels of alanine transaminase and serum creatinine, indicating potential liver and kidney injury. An unusual finding was the 24-day period of continuing positive nucleic acid, which was longer than most patients reported from Wuhan [27].

**Table 1.** Demographic, epidemiologic and clinical characteristics of 788 COVID-19 patients and detailed information on ZJ01 patient.

Characteristics	All patients (% or 1st–3th quarter)	ZJ01 Patient
**Age**	45.83 ± 14.88	30
**Male Sex**	407 (51.65%)	Male
**Current Smoker**	54(6.85%)	No
**Co-existing Condition**		No
Hypertension	126 (15.99%)	
Diabetes	57 (7.23%)	
Chronic liver disease	31 (3.93%)	
Chronic renal disease	7 (0.89%)	
Heart disease	11 (1.40%)	
COPD	3 (0.38%)	
**Date of symptom onset to**		
Outpatient visiting	2 (1–4)	1
PCR confirmation	4 (2–7)	1
Hospital admission	3 (1–6)	1
**Severity on admission**		
Mild	710 (90.1%)	Yes
Severe	61 (7.74%)	
Critical	17 (2.16%)	
**Fever**(yes/no)	636/152(80.71%)	Yes
**Cough**	506/282 (64.21%)	Yes
**Sputum production**	265/523 (33.63%)	Yes
**Sore throat**	111/677 (14.09%)	No
**Muscle ache**	91/697 (11.55%)	No
**GI symptoms**	78/700 (9.89%)	No
**Headache**	66/722 (8.38%)	No
**Laboratory test**		
Leucocytes (×10^9^ per L)	4.8 (3.8–6.0)	4.2
Neutrophils (×10^9^ per L)	2.96 (2.22–4.0)	2.5
Lymphocytes (×10^9^ per L)	1.19 (0.9–1.56)	0.9
INR(normal range 0.85–1.15)	1.02 (0.97–1.09)	1.01
ALT (U/L)	21.09 (15.0–33.0)	57
AST (U/L)	25.0 (19.6–33.0)	34
Serum creatinine (μmol/L)	66.0 (55.2–78.0)	108
Creatine kinase (U/L)	69.0 (47.0–107.0)	75
C-reactive protein (mg/L)	8.0 (2.4–21.2)	6.5
Procalcitonin (ng/mL)	0.05 (0.03–0.07)	0.07
**Chest x-ray/CT findings** (yes/no)		
Normal	87/701 (11.04%)	
Unilateral pneumonia	164/624 (20.81%)	
Bilateral pneumonia	296/492 (37.56%)	Yes
Multiple mottling and ground-glass opacity	235/553 (29.82%)	

### Potential evolutionary divergence in SARS-CoV-2 and unique feature of ZJ01

**Figure 1.**
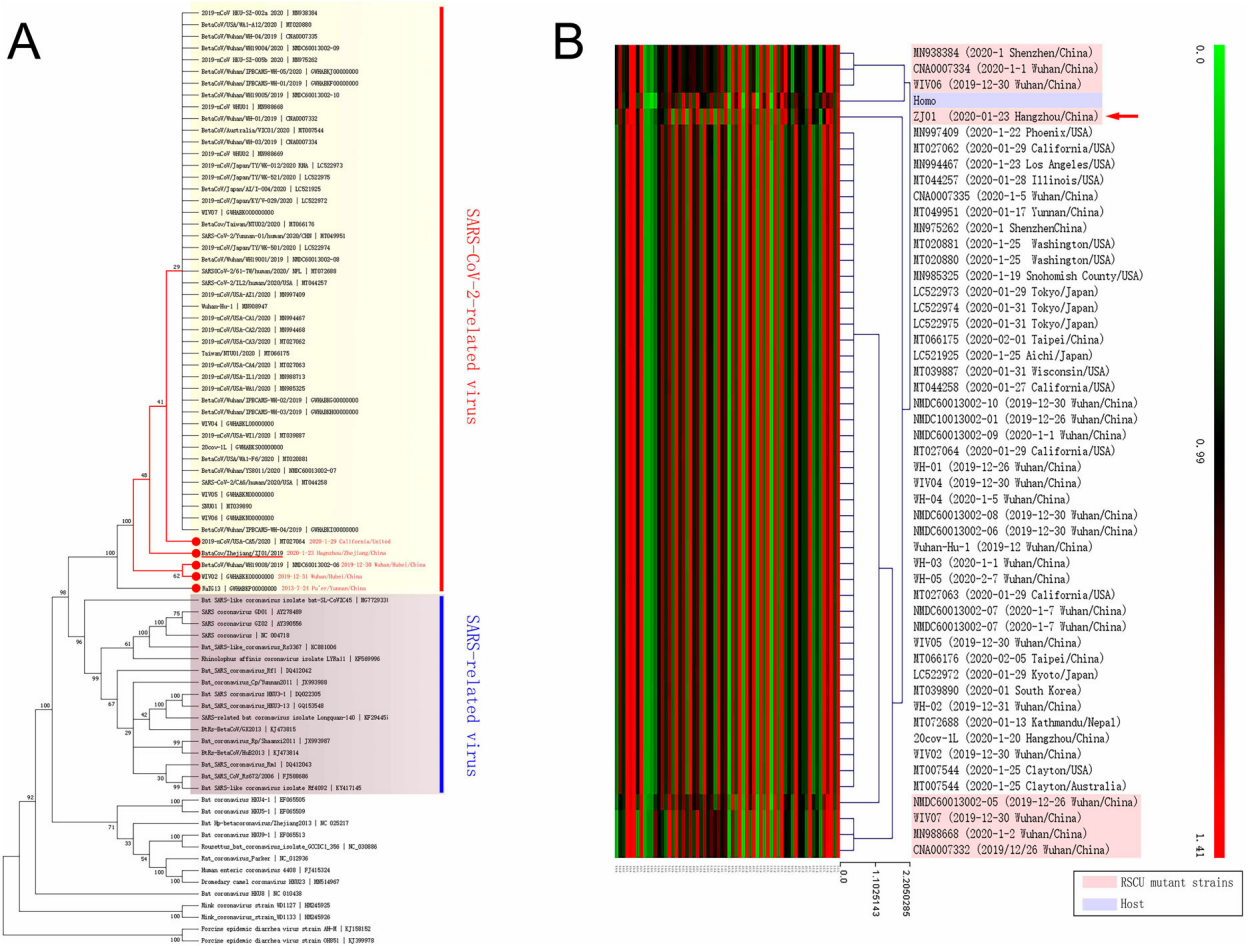
Existence of potential evolutionary divergence in the SARS-CoV-2 family. (A) The data from a phylogenetic analysis indicated that SARS and SARS-CoV-2 are separate branches, while ZJ01 may be representative of a potential evolutionary subtype of the SARS-CoV-2 family. (B) The RSCU heatmap indicates that eight strains of the SARS-CoV-2 family are closer to human RSCU and differ from other strains. ZJ01 might be a representative of potential branches.

Phylogenetic analysis suggested that SARS, RATG13, and SARS-CoV-2 exhibited remarkable evolutionary divergence, with potential evolutionary branches within SARS-CoV-2 (Figure 1(A)). For instance, minor evolutionary divergence existed between WIV02 (2019-12-31)/WH19008 (2019-12-30), and MT0270641 (2020-1-29)/ZJ01(2020-1-23), which were collected in the early and widely disseminated stages of the epidemic, indicating the potential for the formation of evolutionary branches during dissemination. RSCU analysis revealed various differences among the eight strains (MN938384, CNA0007334, WIV06, ZJ01, NMDC60013002-05, CNA0007332, MN988668, and WIV07) and other members of the SARS-CoV-2 family (Figure 1(B)), where MN938384, CNA0007334, WIV06, and ZJ01 were the closest to human RSCU. Among the eight strains collected in Wuhan, Guangdong, and Hangzhou, six were collected at the early stage of COVID-19 (26 December 2019 to 2 January 2020). The collection time of MN938384 was not later than 14 January 2020 (virus submission time), while ZJ01 was collected on 23 January 2020.

### ZJ01 has a novel viral pattern of SARS-CoV-2

**Figure 2.**
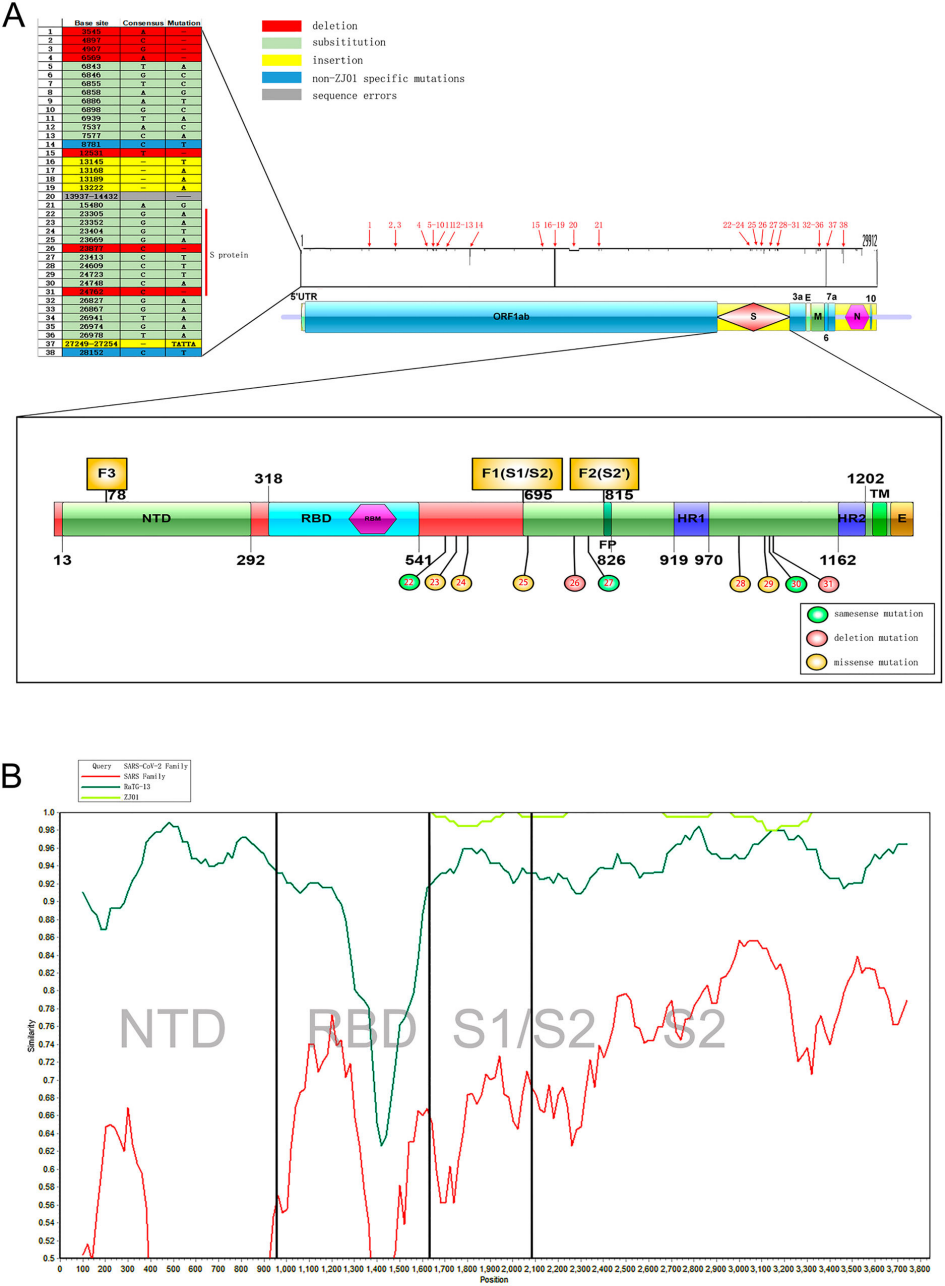
Sequence alignment comparison of ZJ01 mutations with other strains of SARS-CoV-2. (A) The many ZJ01 base mutations are far above average. Some mutations are unique to ZJ01 and appear across every structural domain. Among them, 10 base mutations appear on the S protein, and seven results in amino acid changes. (B) Coronavirus mutations between different species are mainly concentrated in the S1 segment, while the mutations of ZJ01 and other members of SARS-CoV-2 are mainly concentrated in the S2 segment.

The entire sequence of ZJ01 is presented in the Appendix. Sequence alignment analysis indicated 38 mutation sites for ZJ01 compared with other SARS-CoV-2 family members (Figure 2(A)). Of these, 35 mutations were unique to ZJ01, including seven deletions, four insertions, and 24 substitutions. For the remaining three mutation sites, mutation site 20 was caused by a sequencing error, while mutations 14 and 38 are widely distributed in the SARS-CoV-2 family. Among the unique ZJ01 mutations, 10 (mutations 22-31) were located on the S protein. These included three same sense mutations, two deletion mutations, and five missense mutations, which led to amino acid changes of Ser596, Gln613, Glu702, Ala771, Ala1015, Pro1053, and Thr1066. A similarity analysis indicated that the main difference among various coronaviruses located in the receptor-binding domain region of S1. Intriguingly, the differences between ZJ01 and other members of SARS-CoV-2 mainly resided in S2 (Figure 2 (B)). Moreover, 22 mutations resided in an open reading frame segment and five on the M protein. The M glycoprotein is different from other structural proteins in that only a small part of the N-terminus is exposed on the outer surface of the viral envelope. The M protein plays an important role in the process of virus assembly, where it can interact with the N and S proteins. The M protein can specifically interact with viral RNA packaging signals to help RNA form a nucleocapsid.

**Figure 3.**
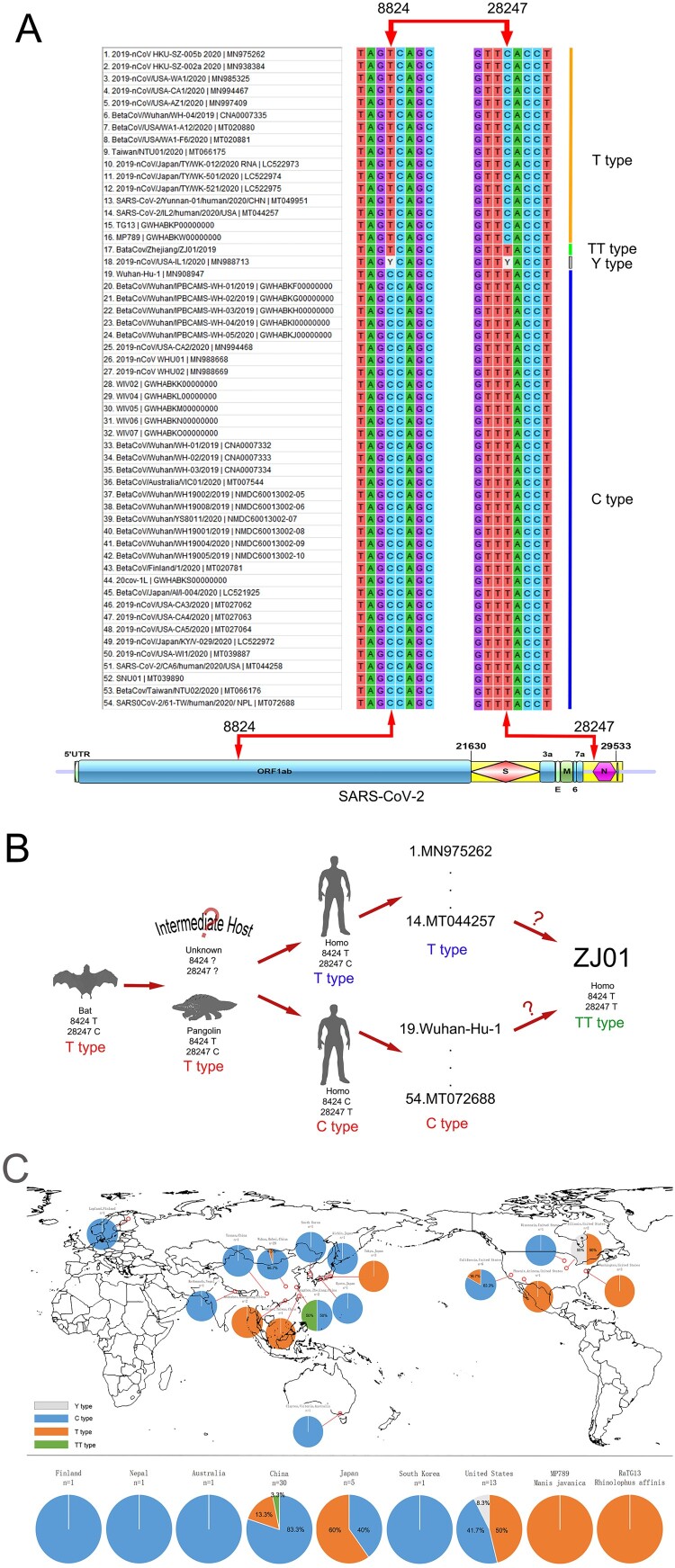
Proposed C/T categorization system for SARS-CoV-2. (A) The SARS-CoV-2 family displayed base mutations in 8824 and 28247 loci with a high (96.3%) correlation. When T appeared in one locus, C would appear in another and vice versa. According to this pattern, we categorized 54 available strains of SARS-CoV-2 into type T (68.5%), type C (27.8%), type TT (1.9%) and type Y (1.9%). (B) BaTG13 and MP789, the possible ancestor of SARS-CoV-2, belong to type T. Therefore, the C/T pattern may appear in the intermediate host or early human transmission stage. (C) Considering global C/T categorization, China, especially the city of Wuhan, presented the C type, while Japan and the United States presented the T type. The difference in the rate of C/T types may help trace the transmission route of SARS-CoV-2.

Further analysis revealed a significant association of gene mutations between nucleotide positions 8824 and 28247 in the SARS-CoV-2 family (Figure 3(A)); when the 8824 was T, its corresponding 28247 was C, and vice versa. This pattern was confirmed in 52 of 54 (96.3%) strains of SARS-CoV-2. The only two exceptions were from ZJ01 (8824T/28247T) and MN988713 (8824Y/28247Y), where Y indicated a C or T. Interestingly, both, Bat coronavirus RATG13 (currently the most similar to SARS-CoV-2) and Pangolin coronavirus MP789 (a suspected intermediate host of SARS-CoV-2) had 8824T/28247C. There was a distance of approximately 20,000 bases between these two nucleotide positions, and no functional correlation was found, where 8824 was in the middle of ORF1ab and 28247 was near the N domain. Since mutation could not explain the perfect correlation between the base change of 8824 and 28247 nucleotide positions in 52 strains of SARS-CoV-2, the most likely possibility was that certain strains of SARS-CoV-2 harbouring 8824C/28247T had a mutation during the early stage of dissemination to become 8824T/28247C. These two ancestor strains kept replicating during the spread of the virus and formed their own cladistics (Figure 3(B)). Although it is still unclear whether 8824T/28247C appeared in the intermediate host stage or at human infection stage, we speculate that SARS-CoV-2 maintained mutation during human transmission and formed the specific strain ZJ01 (8824T/28247T).

We proposed a novel categorization system for SARS-CoV-2 and defined type C as 8824C, type T for 8824T, and type TT for ZJ01 as a special case. According to this system, we further categorized 54 strains of SARS-CoV-2-related viruses (Figure 3(C)). We found a prevalence of the T type of 83.3% in China (*n* = 30) and 95.7% in Wuhan (*n* = 23); 60% of the C type in Japan (*n* = 5) and 100% in Tokyo (*n* = 3); 53.8% of the C type in the United States (*n* = 13), 83.3% of the T type in California (*n* = 6), and 100% of the C type in Washington D.C. (*n* = 3). Intriguingly, for two cases from the US state of Illinois, one was the T type and the other was designated the Y type because of the presence of Y at both nucleotide positions 8824 and 28247, indicating the possibility of co-infection with both T and C types. Worldwide, only one case of the TT type has been found, in Hangzhou. Whether this is an occasional single mutated strain or a novel potential subtype of SARS-CoV-2 warrants more in-depth virologic analysis.

### Evolutionary divergence in SARS-CoV-2 is strongly correlated with Furin protein

**Figure 4.**
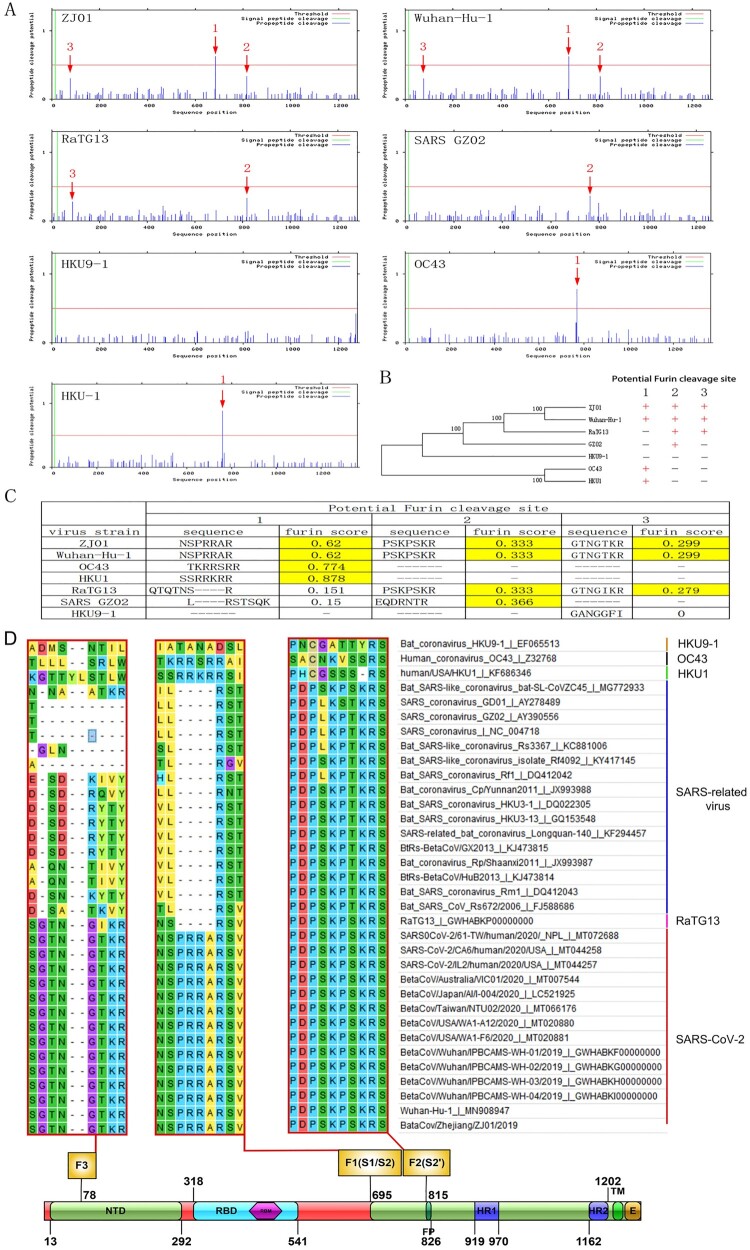
The important role of FCS in SARS-CoV-2 evolution. (A) The number of FCS varied in different coronaviruses. SARS-CoV-2 harboured the F1-3 sites, RaTG13 harboured the F1-2 sites with 96% similarity to SARS-CoV-2, SARS harboured the F2 site, HKU1 and OC43 harboured the F1 site and while HKU9-1 had no FCS. (B) The location and number of FCS may play an important role in coronavirus evolution. (C) The F1 sequence was highly similar among HKU1, OC43 and SARS-CoV-2, perhaps due to translocation of the Furin cleavage site during the evolution of the SARS-CoV-2 ancestor. The result is the ability of human transmission. (D) The extensive conservation of F1-3 in different coronaviruses suggests a potential role in virus invasion and replication.

There were three potential FCSs on the S protein. F1, F2 and F3 were separately located in S1/S2, S2 and the N-terminal domain (NTD) of S1 (Figure 4(A)). Further comparative alignment analysis of GZ02 (SARS viral strain), Wuhan-Hu-1 (the earliest sequenced SARS-CoV-2), RaTG13, HKU9-1 (the potential ancestor of SARS and SARS-CoV-2), HKU-1 and OC43 showed that the variation of FCS sequence had certain regularity in coronavirus evolution (Figure 4(B)). In detail, there was no FCS in HKU9-1, but one potential FCS in the F2 locus of GZ02 (Furin score 0.366) showed effective Furin binding capacity [28]. For RaTG13, the F2 locus was slightly changed (Furin score 0.333) and a novel FCS was formed in the F1 locus (Furin score 0.279). Although the changes in these two sites were inherited in SARS-CoV-2, marked differences in the F1 site between RaTG13 and SARS-CoV-2 were evident.

Strikingly, compared with RaTG13, we found an additional PRRA sequence at the F1 site of SARS-CoV-2 forming a strong and reliable FCS (Furin score 0.62). Although the source of insertion was unknown, the PRRA sequence was common to avian influenza virus [29]. We deduced that it might have been inherited from HKU1 and OC43, which had effective FCS at the F1 site (Furin score 0.878 and 0.744) and the respective amino acid sequence of SSRRKRR and TKRRSRR, with high similarity of NSPRRAR in SARS-CoV-2. HKU1 and CO43 could cause human upper respiratory tract infections, but the symptoms were milder than those caused by SARS and SARS-CoV-2. Epidemiological investigations indicated that OC43 and HKU-1 may be widely present in patients with flu-like symptoms in autumn and winter [30,31]. Coronaviruses may cause co-infection with other respiratory viruses. Therefore, OC43 and HKU-1 are much likely to genetically interact with original SARS-CoV-2. This genetic recombination may have caused the original SARS-CoV-2 to acquire an FCS at the F1 site and eventually become highly infectious and pathogenic (Figure 4(C)). We also found a similar FCS on the S protein of MERS-CoV. Whether this also originated from the genetic recombination of OC43 and HKU-1 is unknown. The source of PRRA on the S protein of SARS-CoV-2 is yet to be confirmed by scientific experiments. The present epidemiological and bioinformatic findings only support speculations. ZJ01 had a Glu702 to Lys702 substitution at amino acid 18 behind the F1 site, and deletion (Ala771 to -) at amino acid 37 ahead of the F2 site. These mutations may influence the tertiary and quaternary structures of the S protein and finally change the Furin binding capacity. The F1-3 sites were conserved in SARS-CoV-2 and SARS (Figure 4(D)), indicating the importance of mutations in these sites.

### Protein structure analyses imply that mutation in the F1 – and f2-related areas of ZJ01 may influence binding with Furin protein

**Figure 5.**
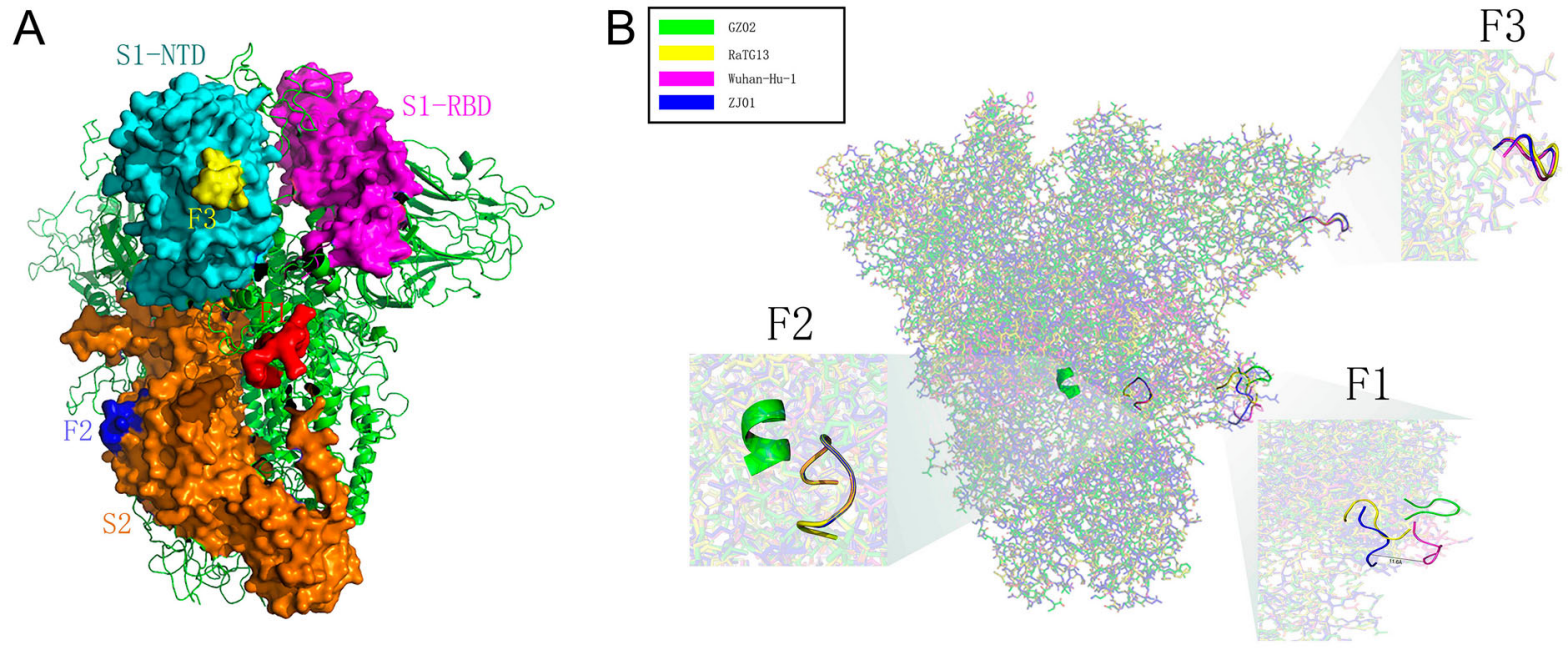
Spatial location and protein structure of potential FCS. (A) The spatial position of F1-3 on S protein. F1 is located at S1/S2, F2 at S2 and F3 at the NTD of S1. (B) Differences in the tertiary structure of the protein at the F1-3 sites of GZ02, RaTG13, Wuhan-Hu-1 and ZJ01. The difference between ZJ01 and Wuhan-Hu-1 may be caused by the mutation of ZJ01 near the FCS.

Homology modelling revealed the position of the F1-3 sites in the S protein of SARS-CoV-2 (Figure 5(A)). F1-3 were located on the surface of S protein and protruded outward, and thus, had great potential as substrate-binding sites. F1 was located in the transition area of S1 and S2 (S1/S2) with an obvious outward protrusion. F2 was located on the mid-lower position of S2, whereas F3 was located on top of S1-NTD. Further homology modelling of the S proteins of GZ02, RaTG13, Wuhan-Hu-1, and ZJ01 revealed significant differences in protein conformation of the F1 locus. From SARS and RaTG13 to SARS-CoV-2, the F1 site showed a tendency towards outward protrusion (Figure 5(B)). Although Wuhan-Hu-1 and ZJ01 shared the same amino acid sequence at the F1 site, the mutation (Glu702 to Lys702) near the F1 site of ZJ01 might have changed its protein conformation and resulted in further outward extension by 11.6 Å. Furthermore, RaTG13, Wuhan-Hu-1, and ZJ01 displayed a high degree of consistency in the F2 site. The F2 site of GZ02 was deeply buried in the inner region of the S protein, which was the biggest difference from SARS-CoV-2, whose F2 site was on the surface of the S protein. Finally, RaTG13, Wuhan-Hu-1, and ZJ01 displayed high similarity at the F3 site that was missing in GZ02.

**Figure 6.**
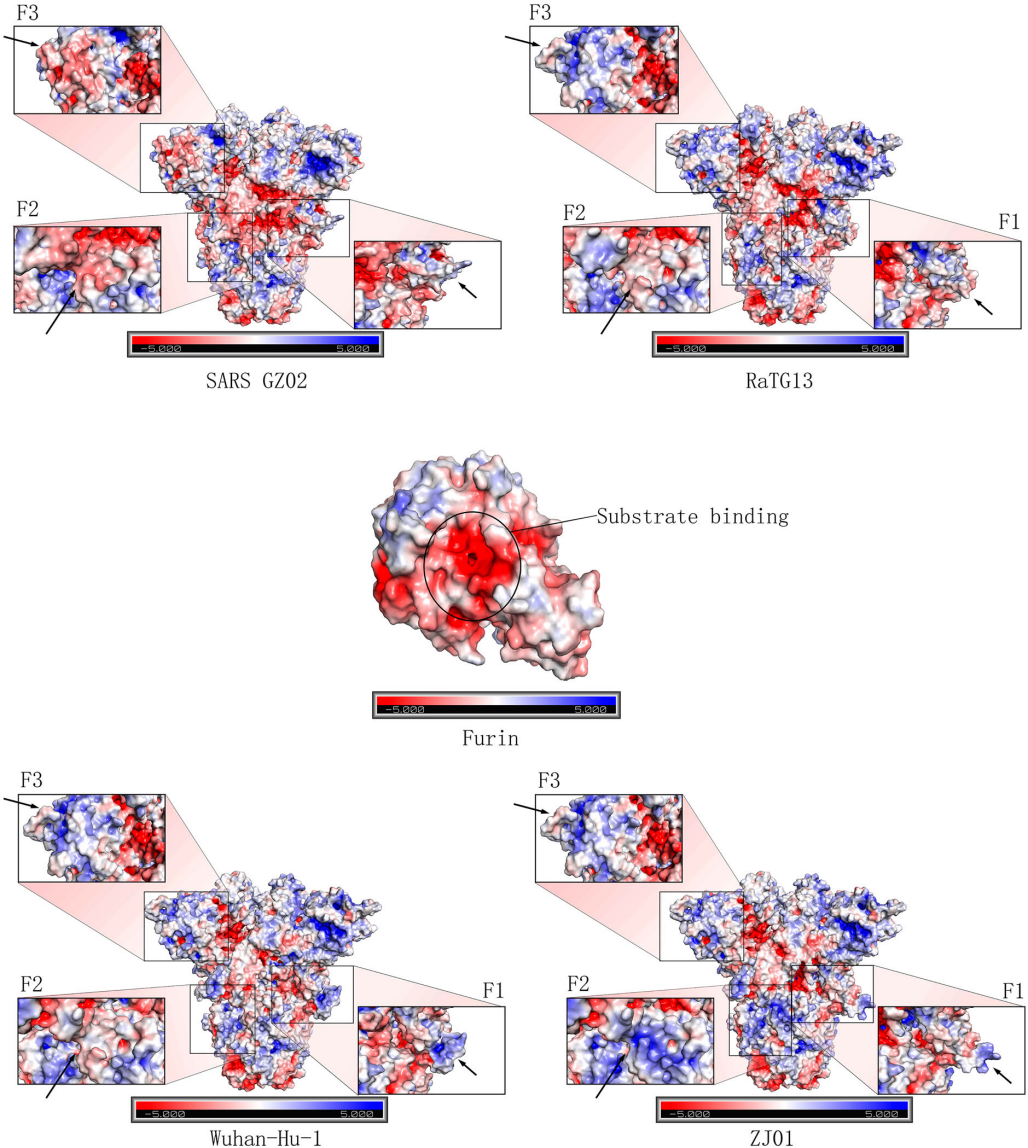
Mutation near the Furin cleavage site may change the surface electrostatic potential of the S protein. Furin has a negative charge, especially in its inner substrate-binding pocket. APBS analysis of the F1-3 sites of GZ02, ZJ01, Wuhan-Hu-1 and RaTG13 revealed a marked difference in electrostatic potential, which may be caused by protein structure changes in F1-3 and nearby loci. Mutation in the F1-2 sites of ZJ01 may alter the binding capacity to Furin, compared with Wuhan-Hu-1.

APBS analysis revealed that Furin was a protease with a negative charge. Its substrate-binding site (191-192, 253–258 and 292-295) was covered with a large number of negative charges (Figure 6). The F1 sites from SARS-CoV-2 related viruses (ZJ01, Wuhan-Hu-1 and RaTG13) were predominantly positively charged, while SARS comprised negative and positive charges. Compared with Wuhan-Hu-1, the F1 site of ZJ01 was more positively charged in its protruding head and more negatively charge in its basal part. The F2 site of GZ02 was covered with a negative charge, whereas the F2 sites of Wuhan-Hu-1 and RaTG13 were covered with a low level of positive charge. The F2 site of ZJ01 was more positively charged than in the other strains, probably due to the nearby gene deletion (Ala 771 to -). GZ02 had many negative charges at the F3 site, while few negative charges were identified in SARS-CoV-2 related virus. We speculated that, despite the gene similarity between ZJ01 and Wuhan-Hu-1, the mutation near the FCS changed the protein structure conformation and surface electrostatic potential of ZJ01, which further influenced its binding capacity with Furin.

### Expression of Furin is greater than ACE2 in different organs at the single-cell level

**Figure 7.**
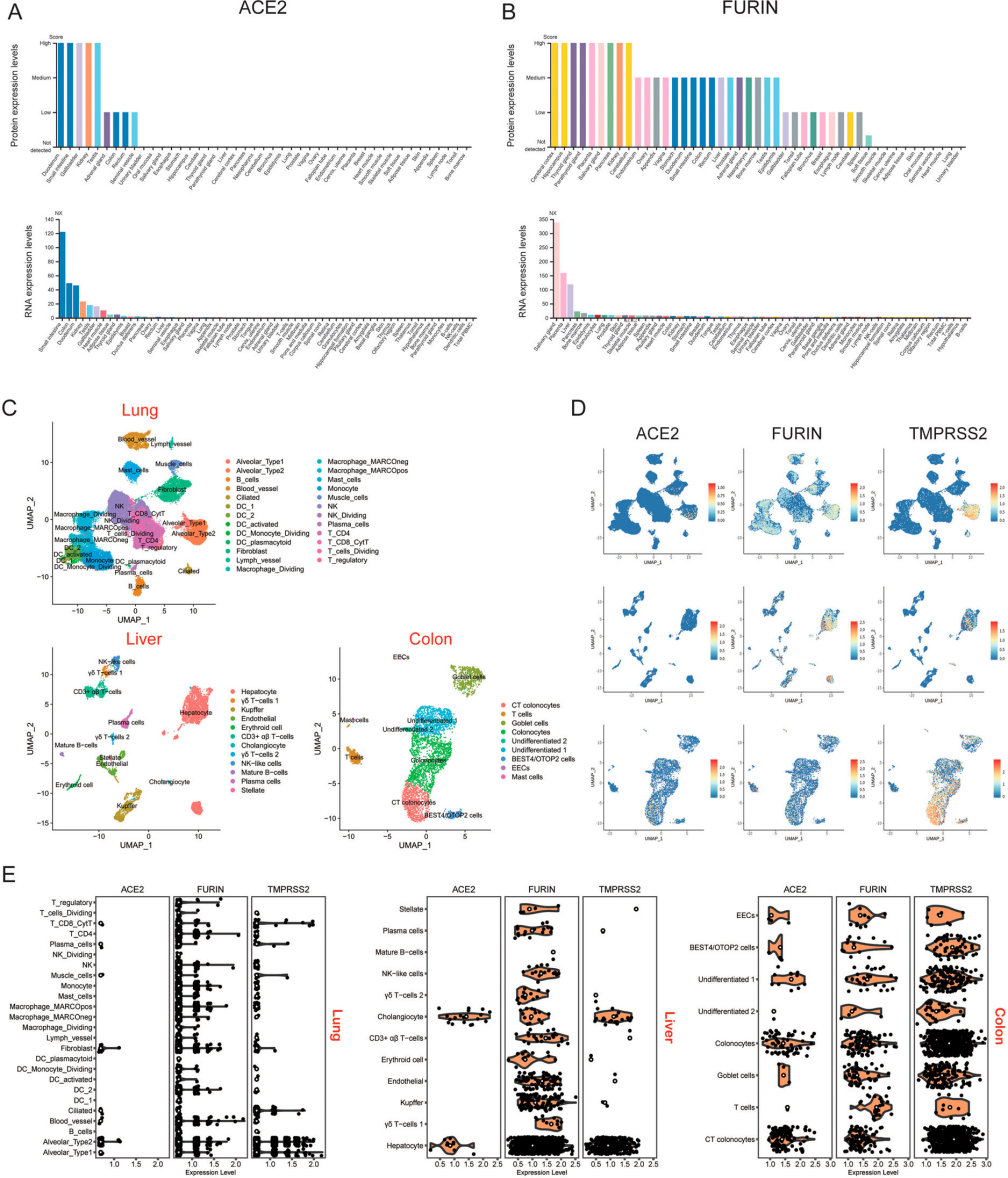
Expression profiles of ACE2 and Furin in multiple human organs. Protein and RNA expression levels of (A) ACE2 and (B) Fusin in major human tissues. NX denotes normalized expression. (C) UMAP plots of cell types in lung, liver and colon scRNA-seq data. Abbreviations are DC, dendritic cells; CT, crypt top; ILCs, innate lymphoid cells; EECs, enteroendocrine cells; BEST4/OTOP2 cells, cells with high expression of BEST4 and OTOP2. (D) UMAP plots indicating the presence of cells that specifically expressed ACE2, Furin and TMPRSS2 in the lung, liver and colon. (E), Violin plots showing ACE2, Furin and TMPRSS2 expression levels in different cell types in lung, liver and colon tissues. Black dots represent the level of gene expression in each cell and white circles represent median values.

The protein and RNA expression levels of ACE2 and Furin in human major tissues were explored in The Human Protein Atlas (https://www.proteinatlas.org/). ACE2 was predominantly expressed in tissues of the small intestine, duodenum, colon, kidneys, and testis, while expression was relatively low in the lung tissue (Figure 7(A)). Furin was expressed in most human tissues and organs, and expression of RNA was highest in the salivary glands, placenta, liver, pancreas, and bone marrow (Figure 7(B)). The expression of the Furin protein was very low in the lungs compared with other tissues.

To further explore the correlation between ACE2 and Furin expression, we reanalysed single-cell RNA sequencing (scRNA-seq) data in the lung, liver, and colon (Figure 7(C)). Since ACE2 and transmembrane protease, serine 2 (TMPRSS2) co-expression have been reported recently [32], we also examined TMPRSS2 expression levels in these tissues. In the scRNA-seq datasets, ACE2, Furin and TMPRSS2 showed higher expression levels in the liver and colon than in the lung (Figure 7(E)). Consistent with a previous report [33], ACE2 was mainly expressed in alveolar type 2 cells in the lungs (Figure 7(D and E)). ACE2 was highly expressed in liver cholangiocytes, liver hepatocytes, colon colonocytes and colon crypt top (CT) colonocytes compared with other liver or colon cell types. This expression pattern was the same as TMPRSS2, but the expression of TMPRSS2 was higher in each cell type. In contrast, Furin was expressed in all cell types of the three tissues, with little co-expression with ACE2.

CD147 (Basigin), a newly identified SARS-CoV-2 receptor, can bind to spike proteins and mediates viral invasion [34]. Recently, elevated plasmin was reported in COVID-19 with comorbidities such as hypertension, diabetes, et al while plasmin or other proteases may be able to cleave FCS [35]. Therefore, we analysed and compared the expression of these genes in the lungs, liver, and colon. We found that CD147, the plasma precursor plasminogen, trypsin, and cathepsin displayed similar expression patterns as ACE2 and TMPRSS2 (Supplementary Fig. 2). However, the expression levels of plasminogen in the lungs and colon were very low under physiological conditions. These results suggest that Furin and other proteases may play important roles in increasing the ability of virus to enter host cells by cleaving the FCS of S protein.

## Discussion

COVID-19 rapidly spread throughout China and has causing enormous damage. During the nationwide dissemination, its epidemiological and clinical features changed. Accumulating evidence indicates the appearance of several unique characteristics distinct from cases in Wuhan [8,9,36], including a higher rate of mild disease, lower rate of severe/critical type and mortality, and longer period of nucleic acid positivity [10,11,27,37]. Moreover, the increased transmission route of SARS-CoV-2 has been gradually unmasked, from previous recognition of respiratory transmission to faeces [38] and even tears and conjunctival secretions [39].

However, recently published virus sequencing results [18] demonstrated that the SARS-CoV-2 family members share similar gene sequences, with only a few essential changes. How could the contradicting phenomenon regarding the change of clinical features and the conservation of viral genome homology be explained? To provide clarity, we selected a COVID-19 patient who experienced a mild disease and isolated the causative virus (ZJ01) for comparative analysis. We found 37 gene mutations, of which 35 were unique to ZJ01. Further bioinformatics analysis highlighted the difference between ZJ01 and other strains of SARS-CoV-2, as well as the important roles of Furin. Thus, we conclude that SARS-CoV-2 may be evolving in a milder direction with increased FCSs.

Analysis of 788 COVID-19 patients in Zhejiang province revealed mild and severe types of SARS-CoV-2. Although we do not currently have evidence to prove whether patients with mild COVID-19 are directly affected by virus mutation or other factors, we found a significant difference between ZJ01 and other members of SARS-CoV-2. ZJ01 had a relatively high number of 37 mutations, and its RSCU was closer to humans than most members of SARS-CoV-2. More importantly, ZJ01 was the only TT type of the 54 strains in our C/T categorization system. Although the sequence of ZJ01 was still close to Wuhan-Hu-1 (the earliest identified SARS-CoV-2) and its mutations were not sufficient to reach the threshold of forming an independent subtype, our evidence indicates that ZJ01 may represent a specific evolutionary direction of SARS-CoV-2.

In this study, we developed the C/T categorization system for SARS-CoV-2, which revealed the occurrence of possibly inheritable mutations at the very early stage of its evolution and the potential for continuing C/T subtype formation. The TT type ZJ01 was unique in our system. Although a similar categorization system has been recently proposed [40], the authors did not report a TT type in their 120 strains of SARS-CoV-2. In addition, the C/T pattern could also be used to trace the route of virus infection and evolution. For instance, we found eight T type strains with 29198T, including RaTG13, MP789, 2019-nCoV_HKU-SZ-005b_2020, and 2019-nCoV_HKU-SZ-002a_2020 from Shenzhen (China), 2019-nCoV/USA-AZ1/2020 from Phoenix, Arizona in the USA, 2019-nCoV/Japan/TY/WK-501/2020, 2019-nCoV/Japan/TY/WK-012/2020 and 2019-nCoV/Japan/TY/WK-521/2020 from Tokyo, Japan. The other 43 strains of SARS-CoV-2 harboured 29198C. Since RaTG13 and MP789 both had 8824C/28247T/29198T, we can speculate that these eight strains of 29198T appeared earlier than strains of 29198C. Using this method, we reckoned that the earliest strain of SARS-CoV-2 infected one patient admitted to Shenzhen hospital on 10 January 2020. The type was 8824C/28247T/29198T/ 2682C/3812C/9606C/11125G/15667T/29808G. The earliest strain in the USA was the aforementioned strain from Phoenix on 26 January 2020 (type was 8824C/28247T/29198T/2682C/3812C/9606T/11125G/15667C/29808G). Patients with these two earliest strains had an exposure history in Wuhan. Therefore, we speculated that the origin of SARS-CoV-9 remains Wuhan, but that the source of the earliest predecessor virus remains vague due to lack of sufficient samples (Supplementary Fig. 1).

Furin is a well-recognized and important serine protease, which has a minimum enzyme restriction site of Arg(R)-X-X-Arg (R). Furin is essential in influenza infection. The binding capacity change of Furin in avian influenza may influence its pathogenicity [41]. Although Furin is not the most common protease in coronaviruses, previous studies have indicated its pivotal roles in SARS and MERS [28,42]. RaTG13 is the closest strain to SARS-CoV-2 with 96% sequence similarity [43]. However, SARS-CoV-2 has a highly conserved PRRA insertion at amino acid 690 amino acid of the S protein, with high conservation [44]. This insertion may become a critical point for the animal-to-human change of the host of RaTG13. Sequence alignment revealed that this inserted sequence may arise from the translocation between human coronavirus HKU1 and OC43 (Figure 4).

SARS-CoV-2 harbours three FCS (F1-3). F1 hydrolyses S protein to S1 and S2 and promotes virus-cell fusion. F2 hydrolyses S2 and participates in virus pathogenicity after cell entry. F3 functions through NTD and promotes adhesion between the virus and cell surface. However, whether the F3 site really exists and, if it does, what its’ function is needs further investigation. Furthermore, the target cell binding site of HKU1 and OC43 was on the S^A^ segment of the S protein, while its corresponding site in SARS-CoV-2 was NTD. Therefore, except for the potential interaction at the F1 site, there also exists the possibility of interaction in the NTD segment between SARS-CoV-2 and HKU1/OC43.

Viruses frequently undergo mutation and adjust their RSCU under evolutionary selection pressure to adapt to the host, thereby acquiring better replication and dissemination capacity [45]. The FCS might be an outstanding marker for coronavirus evolution. Although these three FCSs are very conservative during the evolution of the SARS-CoV-2 family, the evolution of FCS seems to proceed in a different way. We found that although the FCSs of ZJ01 and WuHan-Hu-1 were identical, the mutations of ZJ01 near the F1 and F2 sites changed their three-dimensional protein structure and APBS significantly (Figures 5 and 6). This change is due to the substitution or deletion of amino acids 25–27 on the peptide chain (Figure 2). Although the FCS itself has not changed, the changes in the spatial structure and electrostatic potential of FCS are likely to cause a significant change in the ability of Furin to digest. Therefore, the difference between the SARS-CoV-2 family and other coronavirus families is mainly reflected in the FCS structure. The differences within the SARS-CoV-2 family are likely to be reflected in FCS function. The collective data indicate that Furin plays a pivotal role in the pathogenicity of SARS-CoV-2. The evolutionary trend of increasing FCS in SARS-CoV-2 observed in this study is more prone to influenza-like clinical manifestations, such as human HKU1 and OC43 [46].

Single-cell sequencing analysis revealed a higher expression level and wider organ distribution of Furin than ACE2, especially in the salivary glands, lachrymal glands, colon, liver, and kidneys. Therefore, SARS-CoV-2 might evolve to utilize this specific feature by increasing FCS to become more infectious at multi-organ levels. Our hypothesis is consistent with changes in the clinical characteristics of COVID-19 from published data and our observations, including detection of virus in faeces [38] and conjunctival secretions [39], decreased severity/fatality, increased liver/kidney damage and symptoms of the gastrointestinal tract, increased transmissibility, and prolonged period of nucleic acid positivity. Since ACE2 expression was quite low in the whole body, including the lungs, we speculate that on one hand, the inflammatory reaction rather than the viral load may trigger the severe respiratory damage; on the other hand, the utilization of Furin may help the virus disseminate from the lungs to other organs, leading to decreased severity but increased liver/kidney dysfunction. These speculations must be investigated further. The transmissibility and tropism of SARS-CoV-2 must also be carefully considered.

In summary, ZJ01 isolated from a patient in Zhejiang province with mild COVID-19 patient represents a potential branch in virus evolution. SARS-CoV-2 may adopt a similar mechanism that depends on Furin for invasion as do HJU1 and OC43. Such a potential change in evolutionary direction may promote the appearance of a mild subtype of COVID-19.

## Data Availability

Please see the sequence of ZJ01 in the Appendix of this paper.
